# Participation in Bullying and Associated Health Characteristics, Risk Factors and Leisure Activities: A Profile of School-Age Children in Serbia

**DOI:** 10.3390/ijerph19159159

**Published:** 2022-07-27

**Authors:** Milena Santric-Milicevic, Aleksandar Stevanovic, Nevena Popovac, Filip Milanovic, Suncica Dedovic, Marija Zdravkovic, Nenad Bjelica, Ratko Tomasevic, Jovana Todorovic, Zorica Terzic-Supic, Biljana Obradovic-Tomasevic, Vladimir Milovanovic, Natasa Radosavljevic, Dejan Nikolic

**Affiliations:** 1 Institute of Social Medicine, Faculty of Medicine, University of Belgrade, Dr Subotica, 11000 Belgrade, Serbia; milena.santric-milicevic@med.bg.ac.rs (M.S.-M.); aleksandar.stevanovic@med.bg.ac.rs (A.S.); jovana.todorovic@med.bg.ac.rs (J.T.); zorica.terzic-supic@med.bg.ac.rs (Z.T.-S.); 2 Center-School of Public Health and Health Management, Faculty of Medicine, University of Belgrade, Pasterova 2, 11000 Belgrade, Serbia; 3 University Children’s Hospital, Faculty of Medicine, University of Belgrade, 11000 Belgrade, Serbia; nevena.popovac@gmail.com; 4 Pediatric Surgery Department, University Children’s Hospital, 11000 Belgrade, Serbia; filipmilanovic333@gmail.com; 5 IQVIA, 11000 Belgrade, Serbia; suncica.dedovic@gmail.com; 6 University Hospital Medical Center “Bežanijska kosa”, 11000 Belgrade, Serbia; marija.zdravkovic@med.bg.ac.rs; 7 Faculty of Medicine, University of Belgrade, 11000 Belgrade, Serbia; ratko.tomasevic@med.bg.ac.rs (R.T.); vmilovanovic1972@yahoo.com (V.M.); 8 Primary Health Care Centre “Dr Simo Milosevic”, 11000 Belgrade, Serbia; n.bjelica74@gmail.com; 9 Department of Gastroenterohepatology, Clinic for Internal Medicine, Clinical Hospital Center Zemun, 11000 Belgrade, Serbia; 10 Clinic for Internal Medicine, Rheumatology Outpatient Clinic, Clinical Hospital Center Zemun, 11000 Belgrade, Serbia; biljanatomasevic67@gmail.com; 11 Cardiac Surgery Department, University Children’s Hospital, 11000 Belgrade, Serbia; 12 Department of Biomedical Sciences, State University of Novi Pazar, 36300 Novi Pazar, Serbia; dr.natasa.radosavljevic@gmail.com; 13 Physical Medicine and Rehabilitation Department, University Children’s Hospital, 11000 Belgrade, Serbia

**Keywords:** health characteristics, risk factors, leisure activities, bullying, children

## Abstract

The aim of this study was to examine the prevalence and association of school-age children’s participation in bullying, focusing on their health characteristics, risk factors, and leisure activities. We performed a secondary analysis of the original data of the 2017 HBSC study to examine participation in bullying once and multiple times among school-age children in Serbia. For this purpose, a nationally representative sample of 3267 children from 64 primary and high schools in the Republic of Serbia was evaluated. The outcome variable of interest in our study was participation in bullying. Further groups of individual variables such as health characteristics, risk factors, and leisure activities were assessed. Multivariate regression analysis indicated that children who felt everyday stomach pain, irritability or bad mood, and nervousness were more likely to participate in bullying at least once compared with those who rarely or never had such symptoms by 1.46, 1.58, and 1.58 times, respectively. School-age children who reported being drunk two to three times, and four or more times in life were more likely to participate in bullying than those who reported never being drunk by 1.53 and 1.74 times, respectively. Children who reported to watch TV or other media for five or more hours per day were 2.34 times more likely to be involved in bullying at least once. Multiple regression analysis showed that students with daily stomach pain, back pain, nervousness, and dizziness were more likely to be involved in multiple bullying by 1.16, 1.62, 1.82, and 1.70 times, respectively. Students who had nightly meetings or reported being drunk four or more times in the last 30 days were more likely to be involved in multiple bullying by 2.54 and 3.47, respectively. Students who reported playing games five or more times per day were 2.70 times more likely to be involved in this multiple bullying. This study highlights the importance of professional and family education programmes for early identification of specific health symptoms in the pediatric population, as well as integration with interventions aimed at reducing alcohol abuse among school-age children.

## 1. Introduction

Achievement of Sustainable Development Goal 4 requires addressing a particularly serious problem of physical, psychological, and social bullying in childhood [1], such as pushing and hitting, mocking, leaving people out or ignoring them, as well as other forms of “hurtful and aggressive patterns of behavior perpetrated repeatedly against a less powerful victim” [2]. To help address this issue, World Health Organization (WHO) has published guidelines for managing childhood violence and health system referrals [3]. Indeed, health professionals are among the first contacts in the community for those who seek help and advice. The starting point for strengthening the role of health workers in prevention, and not only in treating poor health outcomes of school-age violence, is to raise community awareness on the common health and behavioral characteristics of children involved in bullying, as well as to design interventions from which they can benefit.

The evidence before this study indicates that the prevalence of various types of bullying varies across regions, and a rough estimate is that every third child globally has been bullied in the past 30 days [4]. According to the meta-analysis of 80 studies in different countries, around 36% of school-age children are bullied by their peers over a period of time [5]. Among different personal and environmental risk factors of peer victimization, the highest levels of social preference, perceived popularity and friendship were associated with a sporadic victimization [5]. Gage et al. showed that school-age children with disabilities and those coming from traditionally marginalized backgrounds were significantly more likely to be involved in bullying [6]. Recent literature underlines the association between bullying in adolescence and developing symptoms of anxiety, depression, and posttraumatic stress over time [7,8]. Bullying marks peer-related factors, e.g., due to power imbalance inside the group, one or more aggressors produce and sustain different types of bullying to harm the victim physically, psychologically, and morally [5,9,10]. A group of authors from Poland and Indonesia found that bullying victimization significantly influences the onset of anxiety in children (panic disorder, generalized anxiety disorder, separation anxiety disorder, and social anxiety) [11]. The manual of mental disorder of the American Psychiatric Association [12] considers bullying as a behavior criterion for the diagnosis of conduct disorder despite considerable distinctions from other conduct problems. However, a recent longitudinal study in a birth cohort of 2232 British children has found an association between bullying behaviors and co-occurring conduct problems based on which it was suggested to tackle bullying behaviors within existing interventions targeting conduct problems and coexisting problems, such as substance use and abuse [13]. Therefore, in-depth knowledge of the targeted population group would be required to design interventions. Furthermore, benefits of the social support (e.g., from family, peers) should be accompanied with counselling services to address the effects of school-aged bullying [7]. These findings were motives for conducting this study to identify the prevalence of bullying and other risky behavior among school-age children in Serbia, and to provide evidence on the necessity for the better integration of anti-bullying programmes and interventions aimed at reducing substance abuse. Additionally, it is essential for health professionals to prevent violence and identify common drivers of violence (e.g., certain characteristics and physical appearance of the child) whenever this is possible [1,4,14]. This is why we also looked for associations between bullying and health and/or leisure characteristics of the children at risk. By investigating health and behavioral characteristics of school-age children partaking in bullying, we want to broaden the knowledge on the prevalence of various forms of bullying and its main predictors in the family and school environment in Serbia [15,16].

The aim of this study was to examine the prevalence of participation in bullying among school-age children and health characteristics, risk factors, and leisure activities of children who participate in it once or multiple times.

## 2. Methods

### 2.1. Study Design and Sample

In this study, we have performed a secondary analysis of the original data of the 2017 Health Behaviour in School-aged Children study (HBSC) in Serbia. For this purpose, the Institute of Public Health of Serbia “Dr Milan Jovanović Batut” (IPHS) granted us an ethical approval (Ethics Committee of IPHS Decision 1934/1, 3 March 2020) to access the database containing the 2017 HBSC data from the nationally representative sample of 3267 students (51.3% were boys and 48.7% girls) from 64 primary (fifth and seventh grade) and high schools (first grade) in the Republic of Serbia (data on the autonomous province of Kosovo and Metohija are not included) [17]. In all four administrative regions of Serbia (Vojvodina, Belgrade, Southern and Eastern Serbia, and Šumadija and Western Serbia), schools were selected using the sampling algorithm that was created by the IPHS to determine the probability proportional to a school’s size and a random number of classes in each school to reach the expected number of students in a sample.

IPHS in cooperation with the World Health Organization (WHO), the Serbian Ministry of Education, Science, and Technological Development, and the Ministry of Health conducted the 2017 HBSC survey following the standardized international HBSC research protocol. Furthermore, IPHS conducted cultural adaptation of the questionnaire, guaranteed informed consent of the participants, and stored the collected HBSC data in its database. It should be noted that schools and parents of the children included in the HBSC study granted approval for children’s participation, and then students voluntary and anonymously completed the questionnaire.

### 2.2. Study Variables

The outcome variable of interest in our study was school-age children participation in bullying. In the survey, there was a specific multiple-choice question: “How often have you been involved in bullying another student during the last few months?”. Possible responses were: None (I have not participated); At least once (once/once or twice); and Multiple times (more than once/twice and more). Therefore, we were able to analyze two variants of the outcome variable—at least one participation in bullying, and multiple participation in bullying. In this study, we focused on individual variables that were described in literature as relevant for understanding the school-age children participation in bullying, including (1) health characteristics, (2) risk factors, and (3) leisure activities. From the Serbian 2017 HBSC database, we created the three sets of variables to be examined as explanatory variables of participation in bullying. The first set of variables included the responses on the health characteristics questions (possible answers/categories are written in the brackets).

- Body Mass Index—BMI (underweight, normal weight, overweight, obese), calculated from the participants information on their weight and height.
- Presence and frequency of several physical and mental symptoms in the last 6 months (rarely or never, almost every week, almost every day). The symptoms included:
  ○ Headache;
  ○ Stomach pain;
  ○ Back pain;
  ○ Depression;
  ○ Irritability or bad mood;
  ○ Nervousness;
  ○ Sleeping problems;
  ○ Dizziness.

- Self-assessed health: “What do you think your health is like?” (Excellent, very good, good, bad).
- Frequency of injuries: “How many times in the last 12 months have you been injured and needed the help of a doctor or nurse?” (I have not been injured in the last 12 months, one time, two times, three times, four and more times).
- Frequency of physical activity: “In the past 7 days, on how many days have you been physically active for at least 1 h?” (None, one to two days, three to four days, five to seven days).
- Intensity of physical activity: “How often do you exercise intensely in your free time so that you lose your breath or sweat?” (Never or rarely, one time per month, one time per week, several times per week/every day).

In the second set of variables, we studied risk behavior including the following:

- Frequency of smoking cigarettes: “How many days (if so) did you smoke cigarettes?”.
  ○ Ever in life? (Never, one to two days, three to five days, six to nine days, 10 or more days).
  ○ During the last 30 days? (I do not smoke, one to two days, three to five days, six to nine days, 10 or more days).

- Smoking status: “How often do you smoke cigarettes now?” (I do not smoke, less than one time per week, at least one time per week but not every day, every day).
- Alcohol abuse: “Have you ever drunk so much alcohol that you were really drunk?”.
  ○ Ever in life? (No, never; Yes, one time; Yes, two to three times; Yes, 4 to 10 times; Yes, more than 10 times).
  ○ During the last 30 days? (No, never; Yes, one time; Yes, two to three times; Yes, four to 10 times; Yes, more than 10 times).

- Quantity of alcohol drinks: “How many alcoholic beverages do you drink during a typical day when you consume alcohol?” (Less than one drink/one drink, two to three drinks, four or more drinks).

The leisure activities were described with the following variables:

- Frequency of daylight meetings with friends: “How often do you meet with friends outside of class—before 8 p.m.?” (Very rarely or never, less than one time per week, every week, everyday).
- Frequency of night time meetings with friends: “How often do you meet with friends outside of class—after 8 p.m.?” (Very rarely or never, less than one time per week, every week, everyday).
- Screen hours per a working day and weekend: “How many hours a day, in your free time, do you usually watch TV, video (including platforms such as YouTube and such), DVD, or some other entertainment on screen?”
  ○ On a working day? (Not at all, up to one hour daily, two to four hours daily, five or more hours daily).
  ○ During the weekend? (Not at all, up to one hour daily, two to four hours daily, five and more hours daily).

- Video gaming hours per a working day and weekend: “How many hours a day, in your free time, do you usually play games on your computer, console, tablet, smartphone, or other device (NOT including games in which you move or practice)?”.
  ○ On a working day? (Not at all, up to one hour daily, two to four hours daily, five and more hours daily).
  ○ During the weekend (Not at all, up to one hour daily, two to four hours daily, five or more hours daily).

- Electronic device hours per a working day and weekend: “How many hours a day, in your free time, do you usually spend with an electronic device, tablet, computer or smartphone for other purposes, e.g., writing homework, sending e-mails, Twitter, Facebook, chat?”.
  ○ On a working day? (Not at all, up to one hour daily, two to four hours daily, five and more hours daily).
  ○ During the weekend (Not at all, up to one hour daily, two to four hours daily, five or more hours daily).

### 2.3. Statistical Analysis

In the study, the prevalence of the outcome variable was calculated as percentages with a 95% confidence interval (CI). With the Pearson chi-square test we were able to assess the statistically significant different variables (at *p* < 0.05). Furthermore, we examined two statistical models of the participation in bullying among school-aged children:

- Model 1: Participating in bullying at least once versus none;
- Model 2: Participating in bullying multiple times versus none.

Both models present strong correlation between the explanatory variables and outcome variables, which was examined using a cross-odds ratio (OR) with a 95% CI of the univariate and multivariate logistic regression. All statistical analyses were performed by the IBM SPSS statistics for windows, version 22.0 (IBM Corp., Armonk, NY, USA).

## 3. Results

### 3.1. Prevalence of Participation in Bullying and Health Variables

Among Serbian school-age children, the prevalence of participation in bullying at least once was almost five times higher in the overweight group of study participants (10.7%) versus those that were underweight (2.3%) (Table 1). Students with stomach pain with a frequency of almost every day were less than twice more prevalent in bullying participation that those who rarely or never had stomach pain (21.0% versus 12.5%). The same applies to those with back pain (almost every day—20.3% versus rarely or never—11.8%) and sleeping problems (almost every day—20.4% versus rarely or never—11.5%). For those who were nervous almost every day, prevalence of participation in bullying was twice higher than among students who rarely or never had such a condition (18.0% versus 9.0%). Presence of everyday depression (19.4%), irritability or bad mood (18.7%), and dizziness (19.5%) was significantly more prevalent in students involved in bullying participation than those that rarely or never had such conditions (12.2%, 10.3% and 12.5% respectively). Students with bad perception of health were twice more prevalent in bullying participation than those with excellent perception of health (28.6% versus 13.4%). Study participants who reported being injured four or more times in the last 12 months with a medical need were more than twice as prevalent in bullying participation than those that had not been injured (29.1% versus 11.0%) (Table 1).

### 3.2. Prevalence of Participation in Bullying and Risk Behavior Variables

The prevalence of participation in bullying was twice higher in students who were actively smoking cigarettes 10 or more days ever in life (25.0%) versus those who never smoked before (12.4%) (Table 2). Participation multiple times in bullying was about three times more frequent for those who were smoking 10 or more days (14.9%) versus those who never smoked (4.4%) or who were smoking one to two days (4.9%). Furthermore, participation in bullying was around three time more frequent for those who were smoking 10 or more days in the last 30 days (18.3%) versus those who did not smoke at all (4.8%). Those who were currently smoking everyday were more than two times more frequently involved in bullying (18.4%) than those who did not smoke cigarettes (8.1%).

Among students who participated in bullying once or multiple times, almost half were drunk more than 10 times in the last month (48.2%), and almost two-fifths were also drunk more than 10 times ever in life (37.2%). Students who were drunk more than 10 times ever in life participated around seven times more often in multiple bullying episodes (26.2%) compared with those who were not drunk at all (3.7%). Students who were drunk more than 10 times during the last 30 days participated about eight times more often in multiple bullying (44.6%) comparing with those who were not drunk at all (5.4%) (Table 2).

### 3.3. Prevalence of Participation in Bullying and Leisure Activities

The prevalence of participation in bullying at least once for students who meet friends before 8 p.m. everyday (9.3%) was about 1.5 times higher than those who very rarely or never meet during day time (6.2%) (Table 3). Students who meet friends after 8 p.m. everyday participated more frequently in bullying at least once (8.9%) or multiple times (8.9%) than those who meet friends very rarely or never in the night time (6.1% and 4.1%, respectively) (Table 3). Participants who watched TV or other multimedia during free time on working days for five or more hours (14.7%) participated in bullying more than twice as often compared with those with no screen time in the same period (6.2%). Participation in multiple bullying for those who watched multimedia five or more hours daily (10.1%) was about 2.5 times more frequent compared with those who watched multimedia up to one hour per day (3.5%). More frequent participation in multiple bullying was seen among those who watched multimedia five or more hours daily (8.8%) compared with those that watched up to one hour daily (3.9%).

Students who played video games five or more hours daily during the working days (11.3%) were almost three times more often involved in multiple bullying than those who never played or played up to one hour per day (3.8%). Similarly, those who played video games five or more hours daily during weekends (12.1%) were more than three times more often involved in multiple bullying compared with those that played games up to one hour daily over the weekend (3.6%).

Participants that spent their free time with electronic devices five or more hours daily during working days (9.4%) were about 2.5 times more often involved in multiple bullying than those who used electronic devices up to one hour per day (3.6%). Students who used electronic devices five or more hours daily during weekends (8.1%) were around two times more often involved in multiple bullying than those who used electronic devices up to one hour daily (3.9%) (Table 3).

### 3.4. Model 1: Participating in Bullying at Least Once

According to univariate regression analysis, certain students’ health characteristics were significantly associated with participating in bullying at least once (Model 1): BMI, headache, stomach and back pain, depression, irritability or bad mood, nervousness, sleeping problems, dizziness, and being injured and in-need of medical help (Supplementary Material Table S1). Participating in bullying at least once (Model 1) was significantly associated with the history of smoking cigarettes and being drunk, as well as meeting with friends outside of class before and after 8 p.m., watching videos and multimedia during working days, and playing games across the entire week (Supplementary Material Table S1).

Multivariate regression analysis (Table 4) indicated that participation in bullying at least once is less likely for school-age children who were underweight (by 77%) than those with normal body weight. On contrary, bullying at least once is more likely for those who felt everyday stomach pain, irritability or bad mood, and nervousness than for those who rarely or never had such symptoms by 1.46, 1.58, and 1.58 times, respectively. Students who had medically treated injuries once/twice or three or more times were more likely to partake in bullying compared with those who were not injured in the last 12 months by 1.51 and 2.06 times, respectively.

School-age children who reported being drunk two to three times in life were by 1.53 times more likely to participate in bullying than those who were never drunk. The risk was even higher for those who were drunk four or more times: they were 1.74 times more likely to have participated in bullying at least once. Those who watch TV or other multimedia for five or more hours per day were 2.34 times more likely to partake in bullying at least once (Table 4).

### 3.5. Model 2: Participating in Bullying Multiple Times

Feeling stomach and back pain, depression, irritability or bad mood, nervousness, dizziness, having sleeping problems, not feeling excellent, as well as being injured and requiring medical assistance present factors which are significantly associated with participating in bullying multiple times (Model 2). Participating in bullying multiple times is also significantly associated with smoking cigarettes and frequency of smoking as well as drunkenness (ever in life and during the last 30 days), as night meetings with friends and playing games throughout the week (Supplementary Material Table S1).

Multiple regression analysis indicated that students having daily stomach pain, back pain, nervousness, and dizziness were more likely to be involved in multiple bullying by 1.16, 1.62, 1.82, and 1.70 times, respectively, On the contrary, those who rated their health as good or bad were 60% or 63% less likely to participate in multiple bullying than those with excellent health. Participants who were injured one or two times in the last 12 months and required medical assistance were 1.54 times more likely to participate in multiple bullying, while this probability was even higher if they were injured three or more times (2.34 times). Students who were drunk four or more times in the last 30 days or who had nightly meetings or played games five or more times a day were 3.47, 2.54, and 2.70 times more likely to be involved in multiple bullying (Table 4).

## 4. Discussion

This study identified the prevalence and the profile of Serbian school-age children who were partaking in bullying one or multiple times. Less than one-third of participants felt bad about their health (28.6%) and were injured more than four times in the last year (29.1%). Furthermore, one-fifth of them felt everyday stomach or back pain and dizziness, depression, and sleeping problems. One-third of participants who participated in multiple bullying smoked cigarettes every day (30%) and the majority of them were drunk more than 10 times in the last month (48.2%). The prevalence of bullying slightly differed among those who meet friends before or after 8 p.m. (16.4% or 17.8%), but almost one-quarter of them had watched multimedia or played games during free time on working days, for five or more hours (24.8% or 23.4%), and less than one-fifth of them had usually spent five or more hours per day with an electronic devices writing homework, sending e-mails, on Twitter, Facebook, and chat, etc.

The profile of bullying perpetrators, who participated in bullying at least once, is based on the evidence of its positive association with ill-health characteristics (almost every day stomach pain and nervousness, as well as being injured once or more times in 12 months with a need for medical assistance, almost every-day irritability or bad mood), being drunk two or more times ever in life, and five or more times watching TV or other multimedia.

Multiple bullying is positively associated with everyday stomach pain, back pain, dizziness, being drunk four or more times during the last 30 days, and meeting friends outside class after 8 p.m. every day and playing games five or more times during working days.

These models inform the necessity of recognizing that bullying might have different forms due to which both physical and psychological symptoms and risky behavior might stand out. For example, direct forms refer to direct confrontation including, but not limited, to physical aggression, threats, etc., while indirect forms include relational manipulation [18]. In addition to this, Peeters et al. described several subtypes of bullies, among which some belong to a popular, socially intelligent group, a popular moderate group, and an unpopular, less socially intelligent group [19].

Our findings showed that everyday stomach and back pain is more likely present in the group of students that were taking part in bullying versus their counterparts. These are confirmed by Allen et al., who stated that the pain and discomfort as situation factors could increase the likelihood of aggression in a general aggression model [20]. Indeed, mice models of Khosravi et al. demonstrated that the mice with a present pain were not just more aggressive, but showed lesser attack latency than the control group [21]. We could add to these findings, i.e., in that the literature distinguishes between the stable and unstable patterns of aggression in children, where perpetrators with high pain tolerance could misperceive pain inflicted by another person, which would disinhibit aggressive action during conflict with others [22]. In this regard, it is necessary to seriously address the physical pain associated with bullying and not ignore it, as it is a signal to act to prevent further bullying from escalating into physical fights among school-aged children.

The results of our study showed that nervousness in children is a significant factor for bullying participation, which is confirmed by the findings of Arslan et al., who indicated that high levels of irritability were associated with bullying [23], which may increase risk of later development of psychotic problems [24,25]. Furthermore, as Hunter et al. [26], we also found that sleeping problems were positively associated with bullying. Therefore, a careful examination of health care practitioners for identification of students susceptible to bullying are advised during the check-ups and screening interventions.

Being injured should be considered from multidimensional aspects. Even though previous reports have stressed that injuries are associated with bullying victimization [27,28,29], it should be pointed out that an injury broadly could be unintentional where older adolescents are particularly susceptible [27] and the ones classified as violent [30]. Additionally, special attention should be given to non-suicidal self-injuries where, aside from bullying history, the other components should be evaluated including the presence of mental illness, childhood abuse, or neglect, etc. [28]. The prevalence of unintentional injuries in low- and middle-income countries is shown to be high in young adolescents [31]. Jansen et al., in their study, found that both the bullied and those who participated in bullying others, among adolescents, reported one to two injuries in recalled time, while the odds increased for both of these categories for adolescents that recall being injured three or more times [29].

Regarding the substance abuse, and in particular alcohol abuse, in the meta-analysis of Vrijen et al. [32], it was pointed out that the bullying perpetration in childhood was positively associated with alcohol use. Furthermore, Loch et al. [33] stressed that bullies were more likely to consume alcohol and to be involved in binge drinking. In the COMPASS study of Williams et al. [34], it was noticed that being a bully at baseline, in the studied student population, was associated with an increase of odds for initiating binge drinking in future. Therefore, it might be assumed that being a bully and alcohol consumption constituting binge drinking have a complex interconnected pattern of action insofar as one might condition the other. Our results demonstrated that children who consume alcohol and were drunk four or more times during the past 30 days have an almost three and a half times higher chance of being involved in multiple bullying occasions as perpetrators. However, having such exposure to alcohol with four or more times being drunk in life was shown to increase the odds of being involved at least once in bullying by more than one and a half times. These findings demonstrate that preventive measures and adequate educational programmes should be implemented in schools as well as primary health centers with parental and/or caregiver active involvement in stressing the importance of possible adverse behavioral patterns and the potential negative influence of alcohol consumption during childhood.

Our study demonstrated that those students who meet friends outside class every day, especially after 8 p.m. (late afternoon and evening), are more than two and a half times likely to participate in multiple fights. These findings might be explained by the increased potential possibility of exposure to the contents, behavioral activities, and interactions that could be associated with increased odds of bullying participation.

In the study of Jolin and Weller, it was noticed that excessive television viewing was associated with sleep, attention, and interpersonal relationship negative effects [35]. Furthermore, the media violence exposure might be associated with aggressive and violent behavior [36]. In addition to this, the study from eight countries of Kuntsche et al. [37] showed that different forms of bullying were associated with television viewing where such an association between television viewing and verbal bullying was consistent across cultures. Moreover, when it comes to the physical bullying, in the same study it was noted that those who practiced a high level of weekend television viewing were associated with this type of bullying [37]. Results of our study are in line to a certain degree with previous reports, where those children who were viewing television five and more hours daily during the weekdays were more than twice likely to participate in at least once in a bullying activity as the perpetrator. Such negative patterns of behavior including bullying have complex multidimensional origins and might be explained by the content-based theories, desensitization to aggression as a consequence of frequent exposure to the violent television programs, and time displacement theories, etc. [36].

Previous studies described the positive association of violent video games and aggression [38,39,40]. Willoughby et al. described the possible theoretical explanations for why playing violent video games could be associated with increased aggression. This includes social learning perspective, excitation transfer theory, a cognitive neoassociation model, and a general aggression model that was adapted from past theories of aggression [38]. However, when it comes to aggression, in the meta-analytic review of Anderson et al. [40], violent video games exposure could increase aggressive behavior, aggressive cognition, aggressive affect, while it could decrease prosocial behavior and empathy. Our findings are consistent with previous reports stating that students who played games five or more hours daily during the working day where more than two and a half times likely to participate in multiple bullying as perpetrators.

There are several limitations to this study. Firstly, it is the absence of the classification of the bullying participants into two distinct groups: those who bully (perpetrators) and those who are being bullied (victims). Thus, further studies should delineate these two categories in order to better describe and understand the potential influence of numerous risk factors that might be associated with bullying behavior as the perpetrator. Furthermore, direct and indirect forms of bullying as well as verbal and physical bullying should be additionally evaluated. Although this cross-sectional study could not identify causation, it pointed out significant correlations between the presence of several psychosomatic symptoms, alcohol drinking, and excessive multimedia use patterns, which are relevant inputs for designing anti-bullying programmes for school-age children in Serbia.

## 5. Conclusions

Profiles of school-age children from Serbia who are involved in bullying once or multiple times most frequently include bad health perceptions, injuries requiring medical assistance, everyday back and stomach pain, everyday nervousness, dizziness, irritability, or bad mood. Additionally, children involved in bullying report everyday television or other multimedia watching, playing games five or more times per day during the working week, and evening meetings with friends.

Our study findings strengthen the WHO recommendations on the need of taking a detailed history to rule out if a poor diet and excessive eating habits are symptoms suggesting bullying as triggering events or stressors and to assess if the injury history is compatible with the bullying. The study also highlighted the importance of the implementation of professional and family education programs for the early recognition of specific harmful health symptoms in the pediatric population, as well as integration with interventions to reduce alcohol abuse among school-age children. This is in line with the WHO guidelines that health professionals should provide counselling messages to parents/caregivers on how to protect children from any form of bullying and in the home, at school, and in the community. It is very important to empower school-age children for an open discussion with their peers, as well as to inform general public about bullying and its prevention strategies in order to support children in becoming independent and assertive individuals.

## Figures and Tables

**Table 1:** Prevalence of bullying among school-age children and their health characteristics, Serbia, 2017.

Health Characteristics	Sample *n* (%)	Participation in Bullying Prevalence (% and 95% CI)			*p*
		At Least Once	Multiple Times	Total	
**Body Mass Index (BMI)**					
Underweight	130 (4.3)	2.3 (−0.3–4.9)	9.2 (4.3–14.2)	11.5 (6.0–17.0)	0.008
Normal weight	2433 (80.5)	8.8 (7.7–9.9)	5.5 (4.6–6.4)	14.3 (12.9–15.7)	<0.001
Overweight	374 (12.4)	10.7 (7.6–13.8)	6.4 (3.9–8.9)	17.1 (13.3–20.9)	0.018
Obese	87 (2.9)	6.9 (1.6–12.2)	4.6 (0.2–9.0)	11.5 (4.8–18.2)	0.257
*p*	<0.001	0.030	0.297	0.396	
Response rate 92.6%					
**How often in the last 6 months have you felt any of the following?**					
**Headache**					
Rarely or never	1806 (57.0)	7.6 (6.4–8.8)	5.3 (4.2–6.3)	12.8 (11.3–14.4)	0.002
Almost every week	527 (16.6)	9.5 (7.0–12.0)	5.5 (3.6–7.4)	15.0 (11.9–18.0)	0.007
Almost every day	834 (26.3)	10.0 (7.9–12.0)	6.2 (4.6–7.9)	16.2 (13.7–18.7)	0.003
*p*	<0.001	0.089	0.595	0.058	
Response rate 96.9%					
**Stomach pain**					
Rarely or never	1507 (47.9)	7.0 (5.7–9.3)	5.6 (4.4–6.7)	12.5 (10.9–14.2)	0.057
Almost every week	1026 (32.6)	8.3 (6.6–10.0)	3.5 (2.4–4.6)	11.8 (9.8–13.8)	<0.001
Almost every day	615 (19.5)	12.4 (9.8–15.0)	8.6 (6.4–10.8)	21.0 (17.8–24.2)	0.016
*p*	<0.001	<0.001	<0.001	<0.001	
Response rate 96.4%					
**Back pain**					
Rarely or never	2053 (65.41)	7.2 (6.1–8.3)	4.6 (3.7–5.5)	11.8 (10.4–13.2)	<0.001
Almost every week	474 (15.1)	11.0 (8.2–13.8)	4.6 (2.7–6.5)	15.6 (12.3–18.9)	<0.001
Almost every day	611 (19.5)	11.0 (8.5–13.4)	9.3 (7.0–11.6)	20.3 (17.1–23.6)	0.172
*p*	<0.001	0.002	<0.001	<0.001	
Response rate 96.1%					
**Depression**					
Rarely or never	2012 (65.4)	7.0 (5.9–8.1)	5.2 (4.2–6.1)	12.2 (10.7–13.6)	0.007
Almost every week	412 (13.4)	9.7 (6.8–12.6)	4.9 (2.8–6.9)	14.6 (11.2–18.0)	0.004
Almost every day	653 (21.2)	12.1 (9.6–14.6)	7.4 (5.3–9.4)	19.4 (16.4–22.5)	0.002
*p*	<0.001	<0.001	0.085	<0.001	
Response rate 94.2%					
**Irritability or bad mood**					
Rarely or never	1077 (34.7)	5.6 (4.2–6.9)	4.7 (3.5–6.0)	10.3 (8.5–12.1)	0.190
Almost every week	759 (24.5)	7.4 (5.5–9.2)	4.2 (2.8–5.6)	11.6 (9.3–13.9)	0.004
Almost every day	1265 (40.8)	11.5 (9.8–13.3)	7.1 (5.7–8.5)	18.7 (16.5–20.8)	<0.001
*p*	<0.001	<0.001	0.007	<0.001	
Response rate 94.9%					
**Nervousness**					
Rarely or never	798 (25.4)	5.3 (3.7–6.8)	3.8 (2.4–5.1)	9.0 (7.0–11.0)	0.074
Almost every week	628 (20.0)	4.8 (3.1–6.4)	4.1 (2.6–5.7)	8.9 (6.7–11.1)	0.292
Almost every day	1715 (54.6)	11.3 (9.8–12.7)	6.8 (5.6–8.0)	18.0 (16.2–19.8)	<0.001
*p*	<0.001	<0.001	<0.001	<0.001	
Response rate 96.1%					
**Sleeping problems**					
Rarely or never	1995 (63.9)	6.9 (5.8–8.0)	4.7 (3.7–5.6)	11.5 (10.1–12.9)	0.001
Almost every week	318 (10.2)	10.1 (6.8–13.4)	3.5 (1.5–5.5)	13.5 (9.8–17.3)	<0.001
Almost every day	807 (25.9)	11.9 (9.7–14.1)	8.6 (6.6–10.5)	20.4 (17.7–23.2)	0.013
*p*	<0.001	<0.001	<0.001	<0.001	
Response rate 95.5%					
**Dizziness**					
Rarely or never	2479 (79.1)	7.8 (6.7–8.8)	4.7 (3.8–5.5)	12.5 (11.2–13.8)	<0.001
Almost every week	301 (9.6)	12.0 (8.3–15.6)	7.6 (4.6–10.6)	19.6 (15.1–24.1)	0.037
Almost every day	353 (11.3)	10.2 (7.0–13.4)	9.3 (6.3–12.4)	19.5 (15.4–23.7)	0.351
*p*	<0.001	0.022	<0.001	<0.001	
Response rate 95.9%					
**What do you think your health is like?**					
Excellent	2081 (65.3)	8.0 (6.8–9.1)	5.4 (4.4–6.4)	13.4 (11.9–14.8)	<0.001
Very good	783 (24.6)	8.6 (6.6–10.5)	5.1 (3.6–6.7)	13.7 (11.3–16.1)	0.003
Good	272 (8.5)	12.5 (8.6–16.4)	5.5 (2.8–8.2)	18.0 (13.4–22.6)	0.002
Bad	49 (1.5)	8.2 (0.5–15.8)	20.4 (9.1–31.7)	28.6 (15.9–41.2)	0.042
*p*	<0.001	0.096	<0.001	<0.001	
Response rate 97.5%					
**How many times in the past 12 months have you been injured and needed the help of a doctor or nurse?**					
I have not been injured in the last 12 months	1840 (57.4)	6.8 (5.7–8.0)	4.1 (3.2–5.0)	11.0 (9.5–12.4)	<0.001
One time	709 (22.1)	9.4 (7.3–11.6)	6.3 (4.6–8.1)	15.8 (13.1–18.5)	0.015
Two times	340 (10.6)	11.8 (8.3–15.2)	6.5 (3.9–9.1)	18.2 (14.1–22.3)	0.008
Three times	136 (4.2)	9.6 (4.6–14.5)	8.1 (3.5–12.7)	17.6 (11.2–24.1)	0.334
Four and more times	179 (5.6)	16.2 (10.8–21.6)	12.8 (7.9–17.8)	29.1 (22.4–35.7)	0.184
*P*	<0.001	<0.001	<0.001	<0.001	
Response rate 99.4%					
**In the past 7 days, how many days have you been physically active for at least 1 h a day?**					
None	159 (5.0)	8.2 (3.9–12.4)	10.1 (5.4–14.7)	18.2 (12.2–24.2)	0.279
One to two days	444 (13.9)	10.1 (7.3–12.9)	4.5 (2.6–6.4)	14.6 (11.4–179)	<0.001
Three to four days	787 (24.6)	7.9 (6.0–9.8)	4.8 (3.3–6.3)	12.7 (10.4–15.0)	<0.001
Five to seven days	1803 (56.5)	8.6 (7.3–9.9)	5.8 (4.7–6.8)	14.4 (12.7–16.0)	<0.001
*p*	<0.001	0.596	0.045	0.297	
Response rate 97.7%					
**How often do you exercise intensely in your free time so that you lose your breath or sweat?**					
Never or rarely	523 (16.7)	9.0 (6.5–11.4)	4.8 (3.0–6.6)	13.8 (10.8–16.7)	0.004
One time per month	187 (6.0)	5.9 (2.5–9.3)	6.4 (2.9–9.9)	12.3 (7.6–17.0)	0.414
One time per week	377 (12.0)	7.7 (5.0–10.4)	4.8 (2.6–6.9)	12.5 (9.1–15.8)	0.049
Several times per week/Every day	2046 (65.3)	9.1 (7.8–10.3)	5.9 (4.9–6.9)	15.0 (13.5–16.6)	<0.001
*p*	<0.001	0.429	0.623	0.459	
Response rate 95.9%					

CI—Confidence interval.

**Table 2:** Prevalence of bullying among school-age children and risk behavior, Serbia, 2017.

Risk Behavior	Sample *n* (%)	Participation in Bullying Prevalence (% and 95% CI)			*p*
		At Least Once	Multiple Times	Total	
**How many days (if so) did you smoke cigarettes?**					
**Ever in life**					
Never	2616 (82.1)	8.0 (7.0–9.0)	4.4 (3.6–5.2)	12.4 (11.1–13.6)	<0.001
One to two days	164 (5.1)	12.2 (7.2–17.2)	4.9 (1.6–8.2)	17.1 (11.3–22.8)	0.009
Three to five days	66 (2.1)	9.1 (2.2–16.0)	9.1 (2.2–16.0)	18.2 (8.9–27.5)	0.286
Six to nine days	26 (0.8)	11.5 (−0.7–23.8)	11.5 (−0.7–23.8)	23.1 (6.9–39.3)	0.342
10 and more days	316 (9.9)	10.1 (6.8–13.5)	14.9 (11.0–18.8)	25.0 (20.2–29.8)	0.036
*p*	<0.001	0.269	<0.001	<0.001	
Response rate 97.6%					
**During the last 30 days**					
I don’t smoke	208 (38.2)	13.9 (9.2–18.6)	4.8 (1.9–7.7)	18.8 (13.4–24.1)	<0.001
One to two days	60 (11.0)	5.0 (−0.5–10.5)	10.0 (2.4–17.6)	15.0 (6.0–24.0)	0.149
Three to five days	32 (5.9)	18.8 (5.2–32.3)	9.4 (−0.7–19.5)	28.1 (12.5–43.7)	0.140
Six to nine days	27 (5.0)	7.4 (−2.5–17.3)	3.7 (−3.4–10.8)	11.1 (−0.7–23.0)	0.276
10 and more days	218 (40.0)	9.6 (5.7–13.5)	18.3 (13.2–23.5)	28.0 (22.0–33.9)	0.004
*p*	<0.001	0.155	<0.001	0.040	
Response rate 95.3%					
**How often do you smoke cigarettes now?**					
I don’t smoke	246 (43.6)	12.2 (8.1–16.3)	8.1 (4.7–11.5)	20.3 (15.3–25.4)	0.968
Less than one time per week	59 (10.5)	10.2 (2.5–17.9)	5.1 (−0.5–10.7)	15.3 (6.1–24.4)	0.149
At least one time per week but not everyday	69 (12.2)	5.8 (0.3–11.3)	7.2 (1.1–13.4)	13.0 (5.1–21.0)	0.365
Everyday	190 (33.7)	11.6 (7.0–16.1)	18.4 (12.9–23.9)	30.0 (23.5–36.5)	0.031
*p*	<0.001	0.498	<0.001	0.067	
Response rate 98.4%					
**Have you ever drunk so much alcohol that you were really drunk?**					
**Ever in life**					
No, never	2251 (70.0)	7.6 (6.5–8.7)	3.7 (2.9–4.5)	11.2 (9.9–12.5)	<0.001
Yes, one time	476 (14.8)	11.6 (8.7–14.4)	6.9 (4.7–9.2)	18.5 (15.0–22.0)	0.007
Yes, two to three times	237 (7.4)	8.9 (5.2–12.5)	4.6 (2.0–7.3)	13.5 (9.2–17.9)	0.034
Yes, four to 10 times	106 (3.3)	11.3 (5.3–17.4)	11.3 (5.3–17.4)	22.6 (14.7–30.6)	0.500
Yes, more than 10 times	145 (4.5)	11.0 (5.9–16.1)	26.2 (19.0–33.4)	37.2 (29.4–45.1)	<0.001
*p*	<0.001	0.031	<0.001	<0.001	
Response rate 98.4%					
**During the last 30 days**					
Not ever once	537 (57.9)	10.1 (7.5–12.6)	5.4 (3.5–7.3)	15.5 (12.4–18.5)	0.002
Yes, one time	228 (24.6)	14.5 (9.9–19.0)	9.2 (5.5–13.0)	23.7 (18.0–29.4)	0.041
Yes, two to three times	83 (8.9)	13.3 (6.0–20.5)	16.9 (8.8–24.9)	30.1 (20.3–10.0)	0.257
Yes, four to 10 times	24 (2.6)	8.3 (−2.7–19.4)	20.8 (4.6–37.1)	29.2 (11.0–47.4)	0.110
Yes, more than 10 times	56 (6.0)	3.6 (−1.3–8.4)	44.6 (31.6–57.7)	48.2 (35.1–61.3)	<0.001
*p*	<0.001	0.132	<0.001	0.005	
Response rate 96.3%					
**How many alcoholic beverages do you drink during a typical day when you consume alcohol?**					
Less than one drink/One drink	123 (7.1)	10.6 (5.1–16.0)	9.8 (4.5–15.0)	20.3 (13.2–27.4)	0.416
Two to three drinks	308 (42.9)	12.3 (8.7–16.0)	8.4 (5.3–11.5)	20.8 (16.2–25.3)	0.057
Four and more drinks	287 (40.0)	9.1 (5.7–12.4)	15.3 (11.2–19.5)	24.4 (19.4–29.4)	0.011
*p*	<0.001	0.433	0.025	0.494	
Response rate 98.2%					

CI—Confidence interval.

**Table 3:** Prevalence of bullying among school-age children and leisure activities, Serbia, 2017.

Leisure Activities	Sample *n* (%)	Participation in Bullying Prevalence (% and 95% CI)			*p*
		At Least Once	Multiple Times	Total	
**How often do you meet with friends outside of class—before 8 p.m.?**					
Very rarely or never	504 (15.9)	6.2 (4.1–8.2)	5.6 (3.6–7.6)	11.7 (8.9–14.5)	0.343
Less than one time per week	490 (15.5)	6.5 (4.3–8.7)	4.1 (2.3–5.8)	10.6 (7.9–13.3)	0.044
Every week	1261 (39.9)	9.8 (8.2–11.5)	5.0 (3.8–6.2)	14.8 (12.9–16.8)	<0.001
Everyday	906 (28.7)	9.3 (7.4–11.2)	7.2 (5.5–8.9)	16.4 (14.0–18.9)	0.052
*p*	<0.001	0.023	0.063	0.008	
Response rate 96.8%					
**How often do you meet with friends outside of class—after 8 p.m.?**					
Very rarely or never	782 (24.8)	6.1 (4.5–7.8)	4.1 (2.7–5.5)	10.2 (8.1–12.4)	0.033
Less than one time per week	558 (17.7)	9.5 (7.1–11.9)	5.4 (3.5–7.2)	14.9 (11.9–17.8)	0.004
Every week	1111 (35.3)	9.5 (7.8–11.3)	4.4 (3.2–5.6)	14.0 (11.9–16.0)	<0.001
Everyday	697 (22.1)	8.9 (6.8–11.0)	8.9 (6.8–11.0)	17.8 (15.0–20.6)	0.500
*p*	<0.001	0.047	<0.001	<0.001	
Response rate 96.4%					
**How many hours a day, in your free time, do you usually watch TV, video (including YouTube and the like), DVD, or some other entertainment on screen?**					
**Working day**					
Not at all	177 (5.6)	6.2 (2.7–9.8)	6.8 (3.1–10.5)	13.0 (8.0–17.9)	0.414
Up to one hour daily	1097 (35.0)	6.7 (5.3–8.2)	3.5 (2.4–4.5)	10.2 (8.4–12.0)	<0.001
Two to four hours daily	1534 (48.9)	9.1 (7.6–10.5)	5.7 (4.5–6.8)	14.7 (13.0–16.5)	<0.001
Five and more hours daily	327 (10.4)	14.7 (10.8–18.5)	10.1 (6.8–13.4)	24.8 (20.1–29.4)	0.037
*p*	<0.001	<0.001	<0.001	<0.001	
Response rate 96.0%					
**Weekend**					
Not at all	166 (5.3)	8.4 (4.2–12.7)	6.6 (2.8–10.4)	15.1 (9.6–20.5)	0.266
Up to one hour daily	660 (21.2)	7.1 (5.2–9.1)	3.9 (2.5–5.4)	11.1 (8.7–13.5)	0.006
Two to four hours daily	1593 (51.2)	8.7 (7.3–10.1)	4.3 (3.3–5.3)	13.0 (11.3–14.6)	<0.001
Five and more hours daily	694 (22.3)	10.2 (8.0–12.5)	8.8 (6.7–10.9)	19.0 (16.1–21.9)	0.180
*p*	<0.001	0.248	<0.001	<0.001	
Response rate 95.3%					
**How many hours a day, in your free time, do you usually play games on your computer, console, tablet (eg. iPad), smartphone, or other device (NOT including games in which you move or practice)?**					
**Working day**					
Not at all	967 (30.8)	7.0 (5.4–8.6)	3.8 (2.6–5.0)	10.9 (8.9–12.8)	<0.001
Up to one hour daily	1242 (39.6)	8.6 (7.1–10.2)	3.8 (2.7–4.8)	12.4 (10.6–14.2)	<0.001
Two to four hours daily	704 (22.5)	9.7 (7.5–11.8)	8.7 (6.6–10.7)	18.3 (15.5–21.2)	0.259
Five and more hours daily	222 (7.1)	12.2 (7.9–16.5)	11.3 (7.1–15.4)	23.4 (17.9–29.0)	0.383
*p*	<0.001	0.055	<0.001	<0.001	
Response rate 96.0%					
**Weekend**					
Not at all	874 (28.0)	7.3 (5.6–9.0)	4.3 (3.0–5.7)	11.7 (9.5–13.8)	0.004
Up to one hour daily	989 (31.7)	7.2 (5.6–8.8)	3.6 (2.5–4.8)	10.8 (8.9–12.8)	<0.001
Two to four hours daily	856 (27.4)	9.8 (7.8–11.8)	5.5 (4.0–7.0)	15.3 (12.9–17.7)	<0.001
Five and more hours daily	404 (12.9)	11.9 (8.7–15.0)	12.1 (8.9–15.3)	24.0 (19.8–28.2)	0.457
*p*	<0.001	0.009	<0.001	<0.001	
Response rate 95.6%					
**How many hours a day, in your free time, do you usually spend with an electronic device, tablet computer (e.g., iPad) or smartphone for other purposes, e.g., writing homework, sending e-mails, on Twitter, Facebook, chat?**					
**Working day**					
Not at all	257 (8.2)	7.0 (3.9–10.1)	6.6 (3.6–9.7)	13.6 (9.4–17.8)	0.431
Up to one hour daily	1161 (37.1)	7.8 (6.3–9.4)	3.6 (2.5–4.7)	11.5 (9.6–13.3)	<0.001
Two to four hours daily	1074 (34.3)	9.6 (7.8–11.4)	4.8 (3.6–6.1)	14.4 (12.3–16.5)	<0.001
Five and more hours daily	639 (20.4)	9.5 (7.3–11.8)	9.4 (7.1–11.7)	18.9 (15.9–22.0)	0.462
*p*	<0.001	0.301	<0.001	<0.001	
Response rate 95.8%					
**Weekend**					
Not at all	282 (9.1)	7.8 (4.7–10.9)	6.4 (3.5–9.2)	14.2 (10.1–18.3)	0.256
Up to one hour daily	895 (28.8)	8.2 (6.4–9.9)	3.9 (2.6–5.2)	12.1 (9.9–14.2)	<0.001
Two to four hours daily	1090 (35.1)	8.8 (7.1–10.5)	4.5 (3.3–5.7)	13.3 (11.3–15.3)	<0.001
Five and more hours daily	837 (27.0)	9.6 (7.6–11.5)	8.1 (6.3–10.0)	17.7 (15.1–20.3)	0.151
*p*	<0.001	0.705	<0.001	0.006	
Response rate 95.0%					

CI—Confidence interval; TV—television.

**Table 4:** Models of participation in bullying of school-age children in Serbia, 2017.

Variables	Multivariate Logistic Regression	
	Model 1: At Least Once versus None	Model 2: Multiple Times versus None
	OR (95% CI)	OR (95% CI)
**Health characteristics**		
**Body mass index**		
Normal weight	1	/
Underweight	0.23 * (0.06–0.96)	/
Overweight	1.27 (0.87–1.87)	/
Obese	0.83 (0.35–1.96)	/
**How often in the last 6 months have you felt any of the following?**		
**Headache**		
Rarely or never	1	/
Almost every week	0.97 (0.66–1.41)	/
Almost every day	0.76 (0.53–1.10)	/
**Stomach pain**		
Rarely or never	1	1
Almost every week	0.90 (0.65–1.24)	0.52 (0.34–0.81)
Almost every day	1.46 * (1.01–2.11)	1.16 ** (0.76–1.77)
**Back pain**		
Rarely or never	1	1
Almost every week	1.40 (0.98–1.99)	0.86 (0.51–1.43)
Almost every day	1.24 (0.88–1.77)	1.62 * (1.08–2.41)
**Depression**		
Rarely or never	1	1
Almost every week	0.97 (0.65–1.47)	0.83 (0.48–1.42)
Almost every day	1.04 (0.73–1.50)	0.87 (0.56–1.38)
**Irritability or bad mood**		
Rarely or never	1	1
Almost every week	1.23 (0.81–1.87)	0.74 (0.45–1.21)
Almost every day	1.58 * (1.04–2.40)	0.97 (0.60–1.56)
**Nervousness**		
Rarely or never	1	1
Almost every week	0.85 (0.51–1.42)	1.26 (0.71–2.26)
Almost every day	1.58 * (1.01–2.46)	1.82 * (1.08–3.06)
**Sleeping problems**		
Rarely or never	1	1
Almost every week	1.34 (0.87–2.05)	0.68 (0.35–1.35)
Almost every day	1.36 (0.99–1.88)	1.43 (0.98–2.10)
**Dizziness**		
Rarely or never	1	
Almost every week	0.81 (0.51–1.28)	1.05 (0.63–1.77)
Almost every day	1.26 (0.83–1.92)	1.70 * (1.03–2.79)
**What do you think your health is like?**		
Excellent	/	1
Very good	/	0.51 (0.22–1.18)
Good	/	0.40 * (0.17–0.94)
Bad	/	0.37 * (0.14–0.98)
**How many times in the past 12 months have you been injured and needed the help of a doctor or nurse?**		
I have not been injured in the last 12 months	1	1
One time or Two times	1.51 ** (1.12–2.03)	1.54 * (1.07–2.21)
Three and more times	2.06 ** (1.36–3.11)	2.34 *** (1.46–3.73)
**In the past 7 days, how many days have you been physically active for at least 1 h a day?**		
None	/	/
One to two days	/	/
Three to four days	/	/
Five to seven days	/	/
**How often do you exercise intensely in your free time so that you lose your breath or sweat?**		
Never or rarely	/	/
One time per month	/	/
One time per week	/	/
Several times per week/Every day	/	/
**Risk behavior**		
**How many days (if so) did you smoke cigarettes?**		
**Ever in life**		
Never	1	1
On to two days	1.40 (0.84–2.35)	1.59 (0.37–6.86)
Three to five days	1.35 (0.62–2.94)	1.04 (0.24–4.52)
More than five days	1.14 (0.73–1.79)	0.96 (0.21–5.26)
**During the last 30 days**		
I don’t smoke	/	1
One to two days	/	1.84 (0.42–8.12)
Three to five days	/	2.93 (0.36–23.65)
More than five days	/	2.05 (0.39–10.72)
**How often do you smoke cigarettes now?**		
I don’t smoke	/	1
Less than one time per week	/	0.40 (0.07–2.23)
At least one time per week/ One time per week	/	0.42 (0.09–2.07)
Everyday	/	1.43 (0.38–5.33)
**Have you ever drunk so much alcohol that you were really drunk?**		
Ever in life		
No, never	1	1
Yes, one time	1.06 (0.63–1.78)	0.70 (0.24–2.00)
Yes, two to three times	1.53 * (1.09–2.16)	1.23 (0.43–3.51)
Yes, four and more times	1.74 ** (1.07–2.82)	1.19 (0.35–4.41)
**During the last 30 days**		
Ne, never	/	1
Yes, one time	/	1.41 (0.55–3.64)
Yes, two to three times	/	1.45 (0.43-4.94)
Yes, four and more times	/	3.47 * (1.08-11.13)
**How many alcoholic beverages do you drink during a typical day when you consume alcohol?**		
Less than one drink/One drink	/	/
Two to three drinks	/	/
Four and more drinks	/	/
Leisure activities		
**How often do you meet with friends outside of class—before 8 p.m.?**		
Very rarely or never	1	/
Less than one time per week	0.85 (0.49–4.50)	/
Every week	1.36 (0.85–2.19)	/
Everyday	1.36 (0.80–2.31)	/
**How often do you meet with friends outside of class—after 8 p.m.?**		
Very rarely or never	1	1
Less than one time per week	1.43 (0.92–2.24)	1.59 (0.91–2.77)
Every week	1.29 (0.86–1.94)	1.40 (0.85–2.29)
Everyday	1.08 (0.66–1.77)	2.54 *** (1.57–4.13)
**How many hours a day, in your free time, do you usually watch TV, video (including YouTube and the like), DVD or some other entertainment on screen?**		
**Working day**		
Not at all	1	/
Up to one hour daily	0.97 (0.48–1.94)	/
Two to four hours daily	1.31 (0.66–2.58)	/
Five and more hours daily	2.34 * (1.12–4.86)	/
**Weekend**		
Not at all	/	/
Up to one hour daily	/	/
Two to four hours daily	/	/
Five and more hours daily	/	/
**How many hours a day, in your free time, do you usually play games on your computer, console, tablet (e.g., iPad), smartphone or other device (NOT including games in which you move or practice)?**		
**Working day**		
Not at all	1	1
Up to one hour daily	1.15 (0.71–1.86)	1.34 (0.70–2.56)
Two to four hours daily	0.97 (0.54–1.72)	1.19 (0.91–5.27)
Five and more hours daily	0.94 (0.44–2.03)	2.70 ** (1.30–5.59)
**Weekend**		
Not at all	1	1
Up to one hour daily	0.93 (0.57–1.53)	0.69 (0.35–1.33)
Two to four hours daily	1.31 (0.77–2.26)	0.69 (0.34–1.40)
Five and more hours daily	1.54 (0.78–3.01)	1.39 (0.63–3.06)
**How many hours a day, in your free time, do you usually spend with an electronic device, tablet computer (e.g., iPad) or smartphone for other purposes, e.g., writing homework, sending e-mails, on Twitter, Facebook, chat?**		
**Working day**		
Not at all	/	/
Up to one hour daily	/	/
Two to four hours daily	/	/
Five and more hours daily	/	/
**Weekend**		
Not at all	/	/
Up to one hour daily	/	/
Two to four hours daily	/	/
Five and more hours daily	/	/

O—Odds ratio; CI—Confidence interval; TV—television; * *p* < 0.05; ** *p* < 0.01; *** *p* < 0.001.

## Data Availability

All data are presented in this study. Original data are available on request from IPHS.

## Supplementary Materials

The following supporting information can be downloaded at: https://www.mdpi.com/article/10.3390/ijerph19159159/s1, Table S1. Models of participation in bullying of school-age children in Serbia, 2017.
