# Supplementary material for: Participation in Bullying and Associated Health Characteristics, Risk Factors and Leisure Activities: A Profile of School-Age Children in Serbia
Source: Int J Environ Res Public Health. 2022 Jul 27;19(15):9159. doi: 10.3390/ijerph19159159 (PMC9367782; doi:10.3390/ijerph19159159)
Supplement: Supplementary file 1 [file ijerph-19-09159-s001.zip › ijerph-1820539-supplementary.pdf]

Supplementary material

Table S1. Models of participation in bullying of school-age children in Serbia, 2017.

Variables	Univariate logistic regression	
	**Model 1: At least Once versus None**	**Model 2: Multiple times versus None**
	**OR (95% CI)**	**OR (95% CI)**
**Health characteristics**		
**Body mass index**		
Normal weight	1	1
Underweight	0.25* (0.08-0.81)	1.62 (0.87-3.02)
Overweight	1.26 (0.88-1.80)	1.21 (0.77-1.89)
Obese	0.76 (0.33-1.76)	0.81 (0.29-2.24)
**How often in the last 6 months have you felt any of the following?**		
**Headache**		
Rarely or never	1	1
Almost every week	1.28 (0.91-1.80)	1.07 (0.70-1.65)
Almost every day	1.36* (1.02-1.82)	1.23 (0.87-1.75)
**Stomach pain**		
Rarely or never	1	1
Almost every week	1.18 (0.87-1.59)	0.62 (0.42-0.93)
Almost every day	1.96*** (1.43-2.68)	1.71** (1.20-2.45)
**Back pain**		
Rarely or never	1	1
Almost every week	1.59** (1.14-2.22)	1.06 (0.66-1.71)
Almost every day	1.68** (1.24-2.29)	2.26*** (1.60-3.18)
**Depression**		
Rarely or never	1	1
Almost every week	1.42 (0.98-2.06)	0.97 (0.59-1.58)
Almost every day	1.88*** (1.41-2.52)	1.55* (1.09-2.21)
**Irritability or bad mood**		
Rarely or never	1	1
Almost every week	1.34 (0.92-1.96)	0.90 (0.57-1.42)
Almost every day	2.28*** (1.67-3.12)	1.66** (1.16-2.36)
**Nervousness**		
Rarely or never	1	1
Almost every week	0.91 (0.56-1.41)	1.10 (0.64-1.88)
Almost every day	2.37*** (1.68-3.35)	2.00** (1.32-3.01)
**Sleeping problems**		
Rarely or never	1	1
Almost every week	1.50 (1.00-2.25)	0.76 (0.40-1.44)
Almost every day	1.93*** (1.46-2.54)	2.04*** (1.48-2.82)
**Dizziness**		
Rarely or never	1	1
Almost every week	1.43 (0.98-2.08)	1.78* (1.12-2.84)
Almost every day	1.67** (1.14-2.45)	2.17*** (1.45-3.26)
**What do you think your health is like??**		
Excellent	1	1
Very good	1.33 (0.45-3.99)	0.24** (0.10-0.57)
Good	0.87 (0.30-2.51)	0.21*** (0.10-0.45)
Bad	0.81 (0.28-2.29)	0.22*** (0.11-0.45)
**How many times in the past 12 months have you been injured and needed the help of a doctor or nurse?**		
I have not been injured in the last 12 months	1	1
One time or Two times	1.59** (1.21-2.08)	1.65** (1.18-2.32)

Three and more times	2.29*** (1.57-3.32)	3.07*** (2.00-4.70)
**In the past 7 days, how many days have you been physically active for at least 1 hour a day?**		
None	1	1
One to two days	1.19 (0.62-2.27)	1.83 (1.05-3.19)
Three to four days	0.90 (0.48-1.69)	0.78 (0.48-1.28)
Five to seven days	1.04 (0.56-1.82)	0.82 (0.56-1.20)
**How often do you exercise intensely in your free time so that you lose your breath or sweat?**		
Never or rarely	1	1
One time per month	0.64 (0.33-1.27)	1.32 (0.65-2.69)
One time per week	0.84 (0.52-1.37)	0.98 (0.53-1.83)
Several times per week/Every day	1.03 (0.73-1.44)	1.26 (0.81-1.96)
**Risk behavior**		
**How many days (if so) did you smoke cigarettes?**		
**Ever in life**		
Never	1	1
On to two days	1.61 (0.99-2.63)	1.17 (0.56-2.45)
Three to five days	1.69 (0.79-3.60)	2.30 (0.97-5.47)
More than five days	1.54* (1.06-2.25)	3.89*** (2.73-5.56)
**During the last 30 days**		
I don’t smoke	1	1
One to two days	0.34 (0.10-1.17)	1.99 (0.69-5.74)
Three to five days	1.52 (0.57-4.06)	2.20 (0.57-8.60)
More than five days	0.74 (0.41-1.33)	3.83*** (1.86-7.88)
**How often do you smoke cigarettes now?**		
I don’t smoke	1	1
Less than one time per week	0.78 (0.31-1.97)	0.58 (0.17-2.05)
At least one time per week/ One time per week	0.43 (0.15-1.27)	0.81 (0.29.2.26)
Everyday	1.08 (0.59-1.94)	2.57* (1.42-4.64)
**Have you ever drunk so much alcohol that you were really drunk?**		
**Ever in life**		
No, never	1	1
Yes, one time	1.20 (0.75-1.94)	1.29 (0.68-2.460
Yes, two to three times	1.67** (1.21-2.30)	2.05** (1.35-3.11)
Yes, four and more times	1.90** (1.24-2.92)	6.96*** (4.74-10.21)
**During the last 30 days**		
Ne, never	1	1
Yes, one time	1.60 (1.00-2.54)	1.89* (1.05-3.40)
Yes, two to three times	1.60 (0.79-3.22)	3.78*** (1.89-7.56)
Yes, four and more times	0.73 (0.25-2.11)	10.21*** (5.64-10.49)
**How many alcoholic beverages do you drink during a typical day when you consume alcohol?**		
Less than one drink/One drink	1	1
Two to three drinks	1.17 (0.60-2.30)	0.87 (0.42-1.79)
Four and more drinks	0.90 (0.45-1.83)	1.66 (0.84-3.27)
**Leisure activities**		
**How often do you meet with friends outside of class - before 8 pm?**		
Very rarely or never	1	1
Less than one time per week	1.05 (0.63-1.75)	0.73 (0.40-1.31)
Every week	1.66* (0.10-2.49)	0.93 (0.59-1.48)
Everyday	1.59* (1.04-2.44)	1.37 (0.86-2.16)
**How often do you meet with friends outside of class - after 8 pm?**		
Very rarely or never	1	1
Less than one time per week	1.63* (1.09-2.45)	1.39 (0.83-2.31)
Every week	1.62** (1.14-2.31)	1.12 (0.71-1.77)

Everyday	1.58* (1.07-2.34)	2.37*** (1.53-3.69)
**How many hours a day, in your free time, do you usually watch TV, video (including YouTube and the like), DVD or some other entertainment on screen?**		
**Working day**		
Not at all	1	1
Up to one hour daily	1.05 (0.55-2.03)	0.50 (0.25-0.97)
Two to four hours daily	1.49 (0.79-2.81)	0.85 (0.46-1.60)
Five and more hours daily	2.73** (1.38-5.42)	1.72 (0.86-3.44)
**Weekend**		
Not at all	1	1
Up to one hour daily	0.81 (0.43-1.51)	0.57 (0.27-1.18)
Two to four hours daily	1.01 (0.57-1.80)	0.63 (0.33-1.22)
Five and more hours daily	1.27 (0.70-2.32)	1.39 (0.71-2.71)
**How many hours a day, in your free time, do you usually play games on your computer, console, tablet (e.g., iPad), smartphone or other device (NOT including games in which you move or practice)?**		
**Working day**		
Not at all	1	1
Up to one hour daily	1.25 (0.91-1.71)	1.01 (0.65-1.56)
Two to four hours daily	1.50* (1.05-2.13)	2.47*** (1.62-3.77)
Five and more hours daily	2.01** (1.25-3.24)	3.43*** (2.01-5.84)
**Weekend**		
Not at all	1	1
Up to one hour daily	0.97 (0.68-1.38)	0.83 (0.52-1.32)
Two to four hours daily	1.40 (0.99-1.97)	1.32 (0.85-2.04)
Five and more hours daily	1.89** (1.27-2.81)	3.24*** (2.08-5.05)
**How many hours a day, in your free time, do you usually spend with an electronic device, tablet computer (e.g., iPad) or smartphone for other purposes, eg. writing homework, sending e-mails, on Twitter, Facebook, chat?**		
**Working day**		
Not at all	1	1
Up to one hour daily	1.09 (0.65-1.85)	0.53 (0.30-0.96)
Two to four hours daily	1.38 (0.82-2.33)	0.74 (0.42-1.30)
Five and more hours daily	1.45 (0.84-2.51)	1.51 (0.86-2.65)
**Weekend**		
Not at all	1	1
Up to one hour daily	1.02 (0.62-1.68)	0.60 (0.33-1.08)
Two to four hours daily	1.12 (0.69-1.81)	0.70 (0.40-1.22)
Five and more hours daily	1.28 (0.78-2.09)	1.31 (0.77-2.28)

OR – Odds ratio; CI – Confidence interval; TV – television; *p<0.05; **p<0.01; ***p<0.001.
